# Tetra­aqua­{5-[(pyridin-2-yl­methyl­idene)amino]benzene-1,3-dicarboxyl­ato-κ^2^ *N*,*N*′}nickel(II) tetra­hydrate

**DOI:** 10.1107/S1600536812027122

**Published:** 2012-06-23

**Authors:** Huan-Huan Wang, Guang-He Duan, Lin Wang, Ya-Bo Xie

**Affiliations:** a College of Environmental and Energy Engineering, Beijing University of Technology, Beijing 100124, People’s Republic of China; b Langfang Health Vocational College, Langfang 065001, People’s Republic of China

## Abstract

The title structure, [Ni(C_14_H_8_N_2_O_4_)(H_2_O)_4_]·4H_2_O, contains a mononuclear Ni^II^ complex formed by a chelating bidentate Schiff base and by four Ni-bonded water mol­ecules. The Ni^II^ atom is in a distorted octa­hedral coordination by two N atoms in a *cis* disposition [Ni—N = 2.0753 (16) and 2.1048 (16) Å] and by four water O atoms [Ni—O = 2.0500 (15)–2.0822 (15) Å]. The crystal structure is completed by four further non-coordinating water mol­ecules and all constituents are linked in a three-dimensional manner by an extensive system of 16 O—H⋯O hydrogen bonds.

## Related literature

For related coordination compounds, see: Buffin *et al.* (2004 ▶); Datta *et al.* (2005 ▶); Jiang *et al.* (2007 ▶).
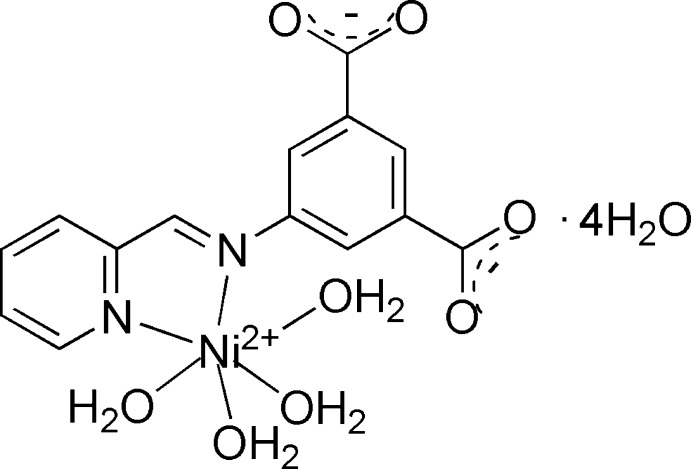


## Experimental

### 

#### Crystal data

[Ni(C_14_H_8_N_2_O_4_)(H_2_O)_4_]·4H_2_O
*M* *_r_* = 471.06
Monoclinic, 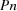

*a* = 10.998 (2) Å
*b* = 7.4536 (15) Å
*c* = 12.271 (3) Å
β = 95.86 (3)°
*V* = 1000.7 (3) Å^3^
*Z* = 2
Mo *K*α radiation
μ = 1.03 mm^−1^
*T* = 153 K
0.28 × 0.25 × 0.23 mm

#### Data collection

Bruker APEXII CCD diffractometer
Absorption correction: multi-scan (*SADABS*; Sheldrick, 2001 ▶) *T*_min_ = 0.76, *T*_max_ = 0.79
4844 measured reflections
3243 independent reflections
3186 reflections with *I* > 2σ(*I*)
*R*_int_ = 0.011

#### Refinement

*R*[*F* ^2^ > 2σ(*F* ^2^)] = 0.018
*wR*(*F* ^2^) = 0.044
*S* = 1.00
3243 reflections
326 parameters
18 restraints
H atoms treated by a mixture of independent and constrained refinement
Δρ_max_ = 0.17 e Å^−3^
Δρ_min_ = −0.32 e Å^−3^
Absolute structure: Flack (1983 ▶), 1482 Friedel pairs>
Flack parameter: 0.012 (7)

### 

Data collection: *APEX2* (Bruker, 2008 ▶); cell refinement: *SAINT* (Bruker, 2008 ▶); data reduction: *SAINT*; program(s) used to solve structure: *SHELXS97* (Sheldrick, 2008 ▶); program(s) used to refine structure: *SHELXL97* (Sheldrick, 2008 ▶); molecular graphics: *SHELXTL* (Sheldrick, 2008 ▶); software used to prepare material for publication: *SHELXTL*.

## Supplementary Material

Crystal structure: contains datablock(s) I, global. DOI: 10.1107/S1600536812027122/qk2036sup1.cif

Structure factors: contains datablock(s) I. DOI: 10.1107/S1600536812027122/qk2036Isup2.hkl

Additional supplementary materials:  crystallographic information; 3D view; checkCIF report

## Figures and Tables

**Table 1:** Hydrogen-bond geometry (Å, °)

*D*—H⋯*A*	*D*—H	H⋯*A*	*D*⋯*A*	*D*—H⋯*A*
O1*W*—H1*WA*⋯O8*W*	0.84 (1)	1.88 (1)	2.715 (3)	176 (3)
O1*W*—H1*WB*⋯O12^i^	0.85 (1)	1.89 (1)	2.739 (2)	177 (3)
O2*W*—H2*WA*⋯O12^ii^	0.85 (1)	1.83 (1)	2.6630 (18)	170 (2)
O2*W*—H2*WB*⋯O14^iii^	0.84 (1)	1.89 (1)	2.719 (2)	170 (2)
O3*W*—H3*WA*⋯O13^iii^	0.84 (1)	1.89 (1)	2.733 (2)	178 (3)
O3*W*—H3*WB*⋯O6*W*	0.85 (1)	1.95 (1)	2.798 (3)	176 (3)
O4*W*—H4*WA*⋯O7*W* ^iv^	0.85 (1)	1.96 (1)	2.751 (2)	156 (2)
O4*W*—H4*WB*⋯O5*W* ^v^	0.84 (1)	1.99 (1)	2.808 (2)	168 (3)
O5*W*—H5*WB*⋯O13	0.84 (1)	1.98 (1)	2.801 (2)	166 (3)
O5*W*—H5*WA*⋯O11^vi^	0.84 (1)	1.96 (1)	2.789 (2)	167 (3)
O7*W*—H7*WB*⋯O13^vii^	0.85 (1)	1.97 (1)	2.797 (3)	165 (4)
O7*W*—H7*WA*⋯O11	0.85 (1)	1.85 (1)	2.685 (2)	167 (3)
O6*W*—H6*WB*⋯O2*W* ^iv^	0.86 (1)	2.42 (4)	3.157 (2)	145 (6)
O6*W*—H6*WA*⋯O7*W* ^iv^	0.86 (1)	1.94 (2)	2.769 (3)	160 (4)
O8*W*—H8*WB*⋯O14^i^	0.84 (1)	2.08 (3)	2.750 (2)	137 (3)
O8*W*—H8*WA*⋯O5*W*	0.85 (1)	2.02 (1)	2.856 (3)	171 (4)

Symmetry codes: (i) 
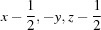
; (ii) 
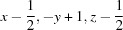
; (iii) 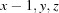; (iv) 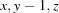; (v) 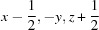; (vi) 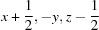; (vii) 
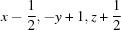
.

**Fig. 1.**
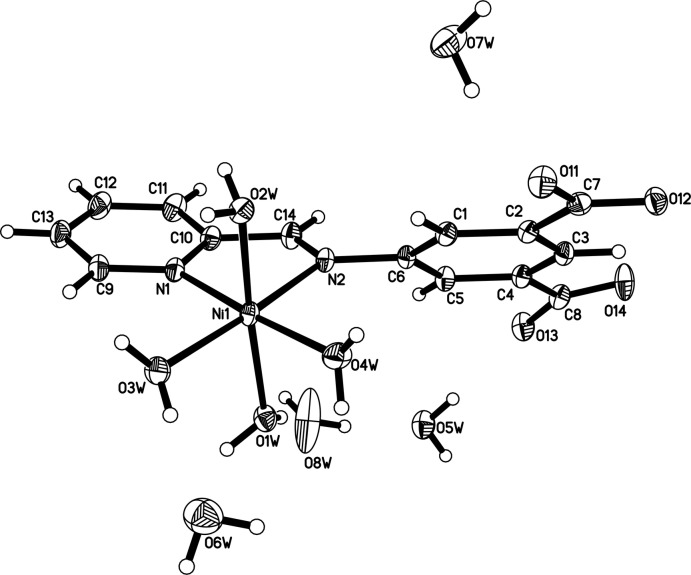
Molecular structure of the title compound with displacement ellipsoids drawn at the 30% probability level for non-hydrogen atoms, hydrogen atoms are shown as small circles of arbitrary radius.

## supplementary crystallographic information

### Comment

Schiff bases with carboxylic groups derived from pyridine 2-carboxaldehyde and amino carboxylic acids are a kind of multifunctional ligand having several potential coordination sites involving N and carboxylate oxygen atoms. The structural chemistry of the coordination compounds of Schiff bases involving some amino benzoic acids and amino acids have been studied (Buffin *et al.*, 2004; Datta *et al.*, 2005; Jiang *et al.*, 2007). However, that of 5-(pyridin-2-ylmethyleneamino)isophthalic acid created by condensation of pyridine 2-carboxaldehyde and 5-aminoisophthalic acid has not been reported. Herein, we describe the synthesis and structural characterization of a new nickel complex coordinated by 5-(pyridin-2-ylmethyleneamino)isophthalic acid. The structure is built up from a neutral mononuclear complex [Ni(C_14_H_8_N_2_O_4_)(H_2_O)_4_] and from four uncoordinated water molecules. Each Ni(II) ion is six-coordinated by two N atoms of the Schiff base ligand and four O atoms from four water molecules, forming a slightly distorted octahedral coordination geometry. The ligand adopts N,N'-bidentate coordination mode with two N atoms chelating Ni(II) in *cis*-configuration. In the complex, the two Ni—N bond lengths differ modestly, with Ni—N (pyridine) = 2.0753 (16) Å and Ni—N (azomethine) = 2.1048 (16) Å. Likewise, the four Ni—O bonds vary little, from 2.0500 (15) to 2.0822 (15) Å. The eight independent water molecules form 16 different 16 O—H···O hydrogen bonds with O···O distances between 2.6630 (18) Å and 3.157 (2) Å (Table 1). They link the constituents into a three-dimensional supramolecular structure. Nine of these hydrogen bonds are accepted by carboxyl oxygen atoms, while seven are accepted by the water molecules (with one exception by non-coordinating water molecules, cf. Table 1).

### Experimental

The title complex was prepared by slowly adding 1 ml of H_2_O solution of 5-(pyridin-2-ylmethyleneamino)isophthalic acid (0.1 mmol, 27.1 mg) to 6 ml of a solution of Ni(NO_3_)_2_ (0.1 mmol, 18.2 mg) in MeOH/H_2_O (V:V = 2:1) at room temperature with stirring. The pH value of the solution was adjusted to 7 by addition of aqueous NaOH. The resulting solution was slowly evaporated at room temperature over several days until green single crystals suitable for X-ray diffraction were obtained.

### Refinement

All C-bound H atoms were placed geometrically and treated as riding with C—H = 0.95 Å and *U*_iso_(H) = 1.2*U*_eq_(C). The water H atoms were located in a difference Fourier map and refined in *x,y,z* and *U*_iso_(H) using an distance restraint of O—H = 0.85 (1) Å.

### Crystal data

**Table:**

[Ni(C_14_H_8_N_2_O_4_)(H_2_O)_4_]·4H_2_O	*F*(000) = 492
*M**_r_* = 471.06	*D*_x_ = 1.563 Mg m^−^^3^
Monoclinic, *P**n*	Mo *K*α radiation, λ = 0.71073 Å
Hall symbol: P -2yac	Cell parameters from 4687 reflections
*a* = 10.998 (2) Å	θ = 2.7–28.3°
*b* = 7.4536 (15) Å	µ = 1.03 mm^−^^1^
*c* = 12.271 (3) Å	*T* = 153 K
β = 95.86 (3)°	Block, green
*V* = 1000.7 (3) Å^3^	0.28 × 0.25 × 0.23 mm
*Z* = 2	

### Data collection

**Table:**

Bruker APEXII CCD diffractometer	3243 independent reflections
Radiation source: fine-focus sealed tube	3186 reflections with *I* > 2σ(*I*)
Graphite monochromator	*R*_int_ = 0.011
π and ω scans	θ_max_ = 25.0°, θ_min_ = 2.4°
Absorption correction: multi-scan (*SADABS*; Sheldrick, 2001)	*h* = −12→13
*T*_min_ = 0.76, *T*_max_ = 0.79	*k* = −8→5
4844 measured reflections	*l* = −14→13

### Refinement

**Table:**

Refinement on *F*^2^	Secondary atom site location: difference Fourier map
Least-squares matrix: full	Hydrogen site location: inferred from neighbouring sites
*R*[*F*^2^ > 2σ(*F*^2^)] = 0.018	H atoms treated by a mixture of independent and constrained refinement
*wR*(*F*^2^) = 0.044	*w* = 1/[σ^2^(*F*_o_^2^) + (0.*P*)^2^] where *P* = (*F*_o_^2^ + 2*F*_c_^2^)/3
*S* = 1.00	(Δ/σ)_max_ = 0.001
3243 reflections	Δρ_max_ = 0.17 e Å^−^^3^
326 parameters	Δρ_min_ = −0.32 e Å^−^^3^
18 restraints	Absolute structure: Flack (1983), 1482 Friedel pairs
Primary atom site location: structure-invariant direct methods	Flack parameter: 0.012 (7)

### Special details

**Table:**

Geometry. All e.s.d.'s (except the e.s.d. in the dihedral angle between two l.s. planes) are estimated using the full covariance matrix. The cell e.s.d.'s are taken into account individually in the estimation of e.s.d.'s in distances, angles and torsion angles; correlations between e.s.d.'s in cell parameters are only used when they are defined by crystal symmetry. An approximate (isotropic) treatment of cell e.s.d.'s is used for estimating e.s.d.'s involving l.s. planes.
Refinement. Refinement of *F*^2^ against ALL reflections. The weighted *R*-factor *wR* and goodness of fit *S* are based on *F*^2^, conventional *R*-factors *R* are based on *F*, with *F* set to zero for negative *F*^2^. The threshold expression of *F*^2^ > σ(*F*^2^) is used only for calculating *R*-factors(gt) *etc*. and is not relevant to the choice of reflections for refinement. *R*-factors based on *F*^2^ are statistically about twice as large as those based on *F*, and *R*- factors based on ALL data will be even larger.

### Fractional atomic coordinates and isotropic or equivalent isotropic displacement parameters (Å^2^)

**Table:**

	*x*	*y*	*z*	*U*_iso_*/*U*_eq_	
Ni1	0.37023 (2)	0.06687 (3)	0.25107 (2)	0.02312 (6)	
O11	0.59391 (13)	0.4492 (2)	0.60907 (11)	0.0365 (3)	
O12	0.79525 (13)	0.44664 (17)	0.65146 (10)	0.0312 (3)	
O13	0.99057 (13)	0.0971 (2)	0.23435 (11)	0.0368 (3)	
O14	1.06809 (13)	0.2502 (2)	0.37992 (11)	0.0437 (4)	
N1	0.33191 (14)	0.1330 (2)	0.08687 (12)	0.0262 (3)	
N2	0.53004 (14)	0.2062 (2)	0.22151 (11)	0.0245 (3)	
C1	0.61826 (18)	0.3092 (3)	0.39979 (14)	0.0251 (4)	
H1	0.5381	0.3345	0.4179	0.030*	
C2	0.71820 (17)	0.3406 (3)	0.47565 (14)	0.0235 (4)	
C3	0.83473 (18)	0.3022 (3)	0.44835 (15)	0.0259 (4)	
H3	0.9034	0.3248	0.5000	0.031*	
C4	0.85227 (17)	0.2312 (3)	0.34669 (14)	0.0250 (4)	
C5	0.75203 (17)	0.1988 (3)	0.27103 (14)	0.0265 (4)	
H5	0.7631	0.1481	0.2017	0.032*	
C6	0.63539 (17)	0.2408 (2)	0.29722 (13)	0.0236 (4)	
C7	0.70072 (18)	0.4171 (2)	0.58770 (15)	0.0262 (4)	
C8	0.97978 (17)	0.1900 (3)	0.31911 (14)	0.0288 (4)	
C9	0.22699 (19)	0.1125 (3)	0.02380 (15)	0.0317 (4)	
H9	0.1610	0.0535	0.0531	0.038*	
C10	0.42487 (17)	0.2153 (3)	0.04433 (14)	0.0276 (4)	
C11	0.4170 (2)	0.2744 (3)	−0.06311 (15)	0.0365 (5)	
H11	0.4852	0.3281	−0.0920	0.044*	
C12	0.3065 (2)	0.2531 (3)	−0.12734 (17)	0.0387 (5)	
H12	0.2975	0.2929	−0.2013	0.046*	
C13	0.2108 (2)	0.1744 (3)	−0.08316 (15)	0.0376 (5)	
H13	0.1339	0.1620	−0.1253	0.045*	
C14	0.53287 (18)	0.2494 (3)	0.12145 (15)	0.0298 (4)	
H27A	0.6038	0.3028	0.0973	0.036*	
O1W	0.45336 (14)	−0.1770 (2)	0.22189 (11)	0.0320 (3)	
H1WA	0.5141 (17)	−0.182 (4)	0.1854 (19)	0.051 (8)*	
H1WB	0.404 (2)	−0.260 (3)	0.197 (2)	0.052 (8)*	
O2W	0.28859 (13)	0.29701 (19)	0.30190 (10)	0.0287 (3)	
H2WA	0.282 (2)	0.380 (2)	0.2549 (15)	0.041 (7)*	
H2WB	0.2170 (12)	0.278 (4)	0.318 (2)	0.040 (7)*	
O3W	0.21080 (13)	−0.0759 (2)	0.25377 (13)	0.0347 (3)	
H3WA	0.1436 (15)	−0.021 (3)	0.2488 (19)	0.045 (7)*	
H3WB	0.213 (3)	−0.161 (3)	0.2999 (18)	0.052 (9)*	
O4W	0.42066 (14)	0.0135 (2)	0.41328 (11)	0.0354 (3)	
H4WA	0.426 (2)	−0.0947 (16)	0.4340 (18)	0.039 (7)*	
H4WB	0.404 (3)	0.071 (3)	0.4687 (15)	0.053 (8)*	
O5W	0.90461 (16)	−0.2004 (3)	0.10994 (13)	0.0428 (4)	
H5WA	0.960 (2)	−0.278 (4)	0.121 (3)	0.072 (11)*	
H5WB	0.923 (3)	−0.118 (3)	0.1558 (17)	0.056 (8)*	
O6W	0.2147 (2)	−0.3434 (3)	0.41405 (17)	0.0606 (5)	
H6WA	0.2869 (17)	−0.353 (6)	0.448 (3)	0.099 (13)*	
H6WB	0.208 (7)	−0.455 (3)	0.400 (7)	0.24 (3)*	
O7W	0.43501 (19)	0.7089 (3)	0.54059 (15)	0.0513 (5)	
H7WA	0.481 (2)	0.617 (3)	0.554 (2)	0.064 (9)*	
H7WB	0.440 (4)	0.779 (4)	0.596 (2)	0.097 (13)*	
O8W	0.6440 (2)	−0.2071 (5)	0.09886 (15)	0.1086 (11)	
H8WA	0.7214 (10)	−0.215 (5)	0.107 (3)	0.098 (12)*	
H8WB	0.638 (3)	−0.166 (5)	0.0353 (14)	0.088 (11)*	

### Atomic displacement parameters (Å^2^)

**Table:**

	*U*^11^	*U*^22^	*U*^33^	*U*^12^	*U*^13^	*U*^23^
Ni1	0.01730 (11)	0.03003 (11)	0.02190 (9)	0.00037 (11)	0.00138 (7)	0.00049 (11)
O11	0.0299 (8)	0.0466 (9)	0.0337 (7)	0.0084 (7)	0.0070 (6)	−0.0074 (7)
O12	0.0304 (8)	0.0353 (8)	0.0270 (6)	0.0010 (6)	−0.0011 (6)	−0.0065 (6)
O13	0.0244 (8)	0.0506 (9)	0.0361 (7)	0.0039 (7)	0.0062 (6)	−0.0093 (7)
O14	0.0179 (7)	0.0810 (12)	0.0321 (7)	−0.0026 (7)	0.0019 (6)	−0.0121 (7)
N1	0.0212 (8)	0.0314 (9)	0.0253 (7)	0.0028 (7)	−0.0011 (6)	−0.0035 (7)
N2	0.0182 (8)	0.0319 (9)	0.0229 (7)	0.0009 (7)	0.0000 (6)	−0.0014 (7)
C1	0.0198 (9)	0.0295 (10)	0.0264 (8)	0.0006 (8)	0.0039 (7)	0.0011 (8)
C2	0.0226 (10)	0.0261 (10)	0.0219 (8)	0.0010 (8)	0.0019 (7)	0.0015 (7)
C3	0.0204 (10)	0.0306 (11)	0.0261 (9)	−0.0008 (8)	−0.0001 (8)	0.0014 (8)
C4	0.0194 (9)	0.0310 (10)	0.0247 (8)	−0.0003 (8)	0.0034 (7)	0.0007 (7)
C5	0.0215 (10)	0.0356 (10)	0.0226 (8)	0.0001 (8)	0.0032 (7)	−0.0012 (8)
C6	0.0189 (10)	0.0288 (9)	0.0225 (8)	−0.0017 (7)	−0.0001 (7)	0.0009 (7)
C7	0.0261 (11)	0.0250 (9)	0.0278 (9)	0.0033 (8)	0.0043 (7)	−0.0002 (7)
C8	0.0219 (10)	0.0388 (11)	0.0264 (9)	0.0023 (8)	0.0051 (7)	0.0026 (8)
C9	0.0253 (10)	0.0376 (11)	0.0309 (9)	0.0000 (9)	−0.0028 (8)	−0.0037 (9)
C10	0.0231 (10)	0.0347 (11)	0.0250 (8)	0.0037 (8)	0.0029 (7)	−0.0020 (8)
C11	0.0359 (12)	0.0491 (13)	0.0248 (9)	0.0052 (10)	0.0043 (8)	0.0009 (9)
C12	0.0434 (14)	0.0479 (14)	0.0228 (9)	0.0077 (11)	−0.0059 (9)	−0.0006 (9)
C13	0.0358 (12)	0.0424 (12)	0.0316 (10)	0.0039 (10)	−0.0108 (8)	−0.0069 (9)
C14	0.0222 (10)	0.0422 (12)	0.0254 (9)	−0.0029 (8)	0.0037 (7)	0.0021 (8)
O1W	0.0236 (8)	0.0363 (8)	0.0361 (7)	0.0018 (7)	0.0035 (6)	−0.0063 (6)
O2W	0.0253 (8)	0.0324 (8)	0.0290 (7)	0.0004 (6)	0.0058 (6)	0.0038 (6)
O3W	0.0210 (8)	0.0361 (8)	0.0475 (8)	−0.0001 (6)	0.0065 (7)	0.0005 (7)
O4W	0.0477 (9)	0.0341 (8)	0.0247 (7)	0.0011 (8)	0.0043 (6)	0.0034 (7)
O5W	0.0378 (10)	0.0533 (11)	0.0367 (8)	0.0026 (9)	0.0010 (7)	−0.0056 (8)
O6W	0.0602 (13)	0.0528 (11)	0.0698 (11)	0.0011 (10)	0.0114 (10)	−0.0038 (10)
O7W	0.0583 (13)	0.0500 (11)	0.0451 (10)	0.0174 (10)	0.0023 (9)	0.0078 (9)
O8W	0.0349 (12)	0.256 (4)	0.0353 (10)	0.0051 (16)	0.0066 (8)	−0.0276 (14)

### Geometric parameters (Å, º)

**Table:**

Ni1—O4W	2.0500 (15)	C9—H9	0.9500
Ni1—O3W	2.0542 (15)	C10—C11	1.384 (3)
Ni1—O2W	2.0621 (14)	C10—C14	1.463 (3)
Ni1—N1	2.0753 (16)	C11—C12	1.389 (3)
Ni1—O1W	2.0822 (15)	C11—H11	0.9500
Ni1—N2	2.1048 (16)	C12—C13	1.364 (3)
O11—C7	1.253 (2)	C12—H12	0.9500
O12—C7	1.255 (2)	C13—H13	0.9500
O13—C8	1.265 (2)	C14—H27A	0.9500
O14—C8	1.247 (2)	O1W—H1WA	0.842 (10)
N1—C9	1.331 (2)	O1W—H1WB	0.853 (10)
N1—C10	1.343 (3)	O2W—H2WA	0.846 (10)
N2—C14	1.273 (2)	O2W—H2WB	0.843 (10)
N2—C6	1.433 (2)	O3W—H3WA	0.842 (10)
C1—C2	1.386 (3)	O3W—H3WB	0.850 (10)
C1—C6	1.389 (2)	O4W—H4WA	0.846 (10)
C1—H1	0.9500	O4W—H4WB	0.837 (10)
C2—C3	1.387 (3)	O5W—H5WA	0.841 (10)
C2—C7	1.519 (3)	O5W—H5WB	0.844 (10)
C3—C4	1.387 (3)	O6W—H6WA	0.862 (10)
C3—H3	0.9500	O6W—H6WB	0.855 (10)
C4—C5	1.388 (3)	O7W—H7WA	0.853 (10)
C4—C8	1.508 (3)	O7W—H7WB	0.850 (10)
C5—C6	1.390 (3)	O8W—H8WA	0.849 (10)
C5—H5	0.9500	O8W—H8WB	0.835 (10)
C9—C13	1.385 (3)		
			
O4W—Ni1—O3W	91.75 (7)	O11—C7—C2	117.99 (16)
O4W—Ni1—O2W	87.18 (6)	O12—C7—C2	117.19 (17)
O3W—Ni1—O2W	91.56 (6)	O14—C8—O13	123.82 (17)
O4W—Ni1—N1	175.40 (7)	O14—C8—C4	118.55 (16)
O3W—Ni1—N1	92.85 (7)	O13—C8—C4	117.63 (16)
O2W—Ni1—N1	92.55 (6)	N1—C9—C13	122.2 (2)
O4W—Ni1—O1W	85.27 (6)	N1—C9—H9	118.9
O3W—Ni1—O1W	86.63 (6)	C13—C9—H9	118.9
O2W—Ni1—O1W	172.17 (5)	N1—C10—C11	122.65 (17)
N1—Ni1—O1W	95.14 (6)	N1—C10—C14	115.33 (15)
O4W—Ni1—N2	96.62 (6)	C11—C10—C14	121.90 (18)
O3W—Ni1—N2	170.98 (6)	C10—C11—C12	118.0 (2)
O2W—Ni1—N2	92.22 (6)	C10—C11—H11	121.0
N1—Ni1—N2	78.80 (7)	C12—C11—H11	121.0
O1W—Ni1—N2	90.69 (6)	C13—C12—C11	119.31 (19)
C9—N1—C10	118.40 (16)	C13—C12—H12	120.3
C9—N1—Ni1	127.97 (14)	C11—C12—H12	120.3
C10—N1—Ni1	113.51 (12)	C12—C13—C9	119.36 (19)
C14—N2—C6	118.84 (16)	C12—C13—H13	120.3
C14—N2—Ni1	113.22 (12)	C9—C13—H13	120.3
C6—N2—Ni1	127.68 (11)	N2—C14—C10	118.80 (17)
C2—C1—C6	119.95 (17)	N2—C14—H27A	120.6
C2—C1—H1	120.0	C10—C14—H27A	120.6
C6—C1—H1	120.0	Ni1—O1W—H1WA	120.9 (19)
C1—C2—C3	119.46 (16)	Ni1—O1W—H1WB	114.8 (19)
C1—C2—C7	120.43 (17)	H1WA—O1W—H1WB	107 (3)
C3—C2—C7	120.11 (16)	Ni1—O2W—H2WA	114.6 (16)
C4—C3—C2	120.86 (17)	Ni1—O2W—H2WB	112.4 (18)
C4—C3—H3	119.6	H2WA—O2W—H2WB	105 (2)
C2—C3—H3	119.6	Ni1—O3W—H3WA	119.3 (19)
C3—C4—C5	119.62 (17)	Ni1—O3W—H3WB	115.6 (19)
C3—C4—C8	119.86 (17)	H3WA—O3W—H3WB	112 (3)
C5—C4—C8	120.52 (16)	Ni1—O4W—H4WA	118.7 (16)
C4—C5—C6	119.66 (16)	Ni1—O4W—H4WB	129.1 (19)
C4—C5—H5	120.2	H4WA—O4W—H4WB	105 (2)
C6—C5—H5	120.2	H5WA—O5W—H5WB	106 (3)
C1—C6—C5	120.42 (17)	H6WA—O6W—H6WB	94 (6)
C1—C6—N2	118.58 (16)	H7WA—O7W—H7WB	111 (3)
C5—C6—N2	120.93 (15)	H8WA—O8W—H8WB	97 (3)
O11—C7—O12	124.81 (17)		
			
O3W—Ni1—N1—C9	11.52 (18)	C14—N2—C6—C1	137.78 (19)
O2W—Ni1—N1—C9	−80.16 (18)	Ni1—N2—C6—C1	−48.5 (2)
O1W—Ni1—N1—C9	98.39 (18)	C14—N2—C6—C5	−45.3 (3)
N2—Ni1—N1—C9	−171.92 (19)	Ni1—N2—C6—C5	128.36 (16)
O3W—Ni1—N1—C10	−172.54 (14)	C1—C2—C7—O11	0.5 (3)
O2W—Ni1—N1—C10	95.77 (14)	C3—C2—C7—O11	−179.73 (19)
O1W—Ni1—N1—C10	−85.67 (14)	C1—C2—C7—O12	−178.20 (17)
N2—Ni1—N1—C10	4.02 (13)	C3—C2—C7—O12	1.6 (3)
O4W—Ni1—N2—C14	175.05 (15)	C3—C4—C8—O14	−12.9 (3)
O2W—Ni1—N2—C14	−97.54 (15)	C5—C4—C8—O14	167.08 (18)
N1—Ni1—N2—C14	−5.37 (14)	C3—C4—C8—O13	167.79 (18)
O1W—Ni1—N2—C14	89.73 (15)	C5—C4—C8—O13	−12.3 (3)
O4W—Ni1—N2—C6	1.06 (16)	C10—N1—C9—C13	−0.5 (3)
O2W—Ni1—N2—C6	88.47 (15)	Ni1—N1—C9—C13	175.25 (16)
N1—Ni1—N2—C6	−179.37 (16)	C9—N1—C10—C11	−2.0 (3)
O1W—Ni1—N2—C6	−84.26 (15)	Ni1—N1—C10—C11	−178.38 (15)
C6—C1—C2—C3	−0.4 (3)	C9—N1—C10—C14	174.02 (18)
C6—C1—C2—C7	179.43 (17)	Ni1—N1—C10—C14	−2.3 (2)
C1—C2—C3—C4	−0.6 (3)	N1—C10—C11—C12	2.5 (3)
C7—C2—C3—C4	179.55 (17)	C14—C10—C11—C12	−173.3 (2)
C2—C3—C4—C5	0.2 (3)	C10—C11—C12—C13	−0.5 (3)
C2—C3—C4—C8	−179.80 (19)	C11—C12—C13—C9	−1.9 (3)
C3—C4—C5—C6	1.2 (3)	N1—C9—C13—C12	2.5 (3)
C8—C4—C5—C6	−178.80 (17)	C6—N2—C14—C10	−179.54 (17)
C2—C1—C6—C5	1.8 (3)	Ni1—N2—C14—C10	5.9 (2)
C2—C1—C6—N2	178.69 (16)	N1—C10—C14—N2	−2.5 (3)
C4—C5—C6—C1	−2.2 (3)	C11—C10—C14—N2	173.6 (2)
C4—C5—C6—N2	−179.01 (17)		

### Hydrogen-bond geometry (Å, º)

**Table:**

*D*—H···*A*	*D*—H	H···*A*	*D*···*A*	*D*—H···*A*
O1*W*—H1*WA*···O8*W*	0.84 (1)	1.88 (1)	2.715 (3)	176 (3)
O1*W*—H1*WB*···O12^i^	0.85 (1)	1.89 (1)	2.739 (2)	177 (3)
O2*W*—H2*WA*···O12^ii^	0.85 (1)	1.83 (1)	2.6630 (18)	170 (2)
O2*W*—H2*WB*···O14^iii^	0.84 (1)	1.89 (1)	2.719 (2)	170 (2)
O3*W*—H3*WA*···O13^iii^	0.84 (1)	1.89 (1)	2.733 (2)	178 (3)
O3*W*—H3*WB*···O6*W*	0.85 (1)	1.95 (1)	2.798 (3)	176 (3)
O4*W*—H4*WA*···O7*W*^iv^	0.85 (1)	1.96 (1)	2.751 (2)	156 (2)
O4*W*—H4*WB*···O5*W*^v^	0.84 (1)	1.99 (1)	2.808 (2)	168 (3)
O5*W*—H5*WB*···O13	0.84 (1)	1.98 (1)	2.801 (2)	166 (3)
O5*W*—H5*WA*···O11^vi^	0.84 (1)	1.96 (1)	2.789 (2)	167 (3)
O7*W*—H7*WB*···O13^vii^	0.85 (1)	1.97 (1)	2.797 (3)	165 (4)
O7*W*—H7*WA*···O11	0.85 (1)	1.85 (1)	2.685 (2)	167 (3)
O6*W*—H6*WB*···O2*W*^iv^	0.86 (1)	2.42 (4)	3.157 (2)	145 (6)
O6*W*—H6*WA*···O7*W*^iv^	0.86 (1)	1.94 (2)	2.769 (3)	160 (4)
O8*W*—H8*WB*···O14^i^	0.84 (1)	2.08 (3)	2.750 (2)	137 (3)
O8*W*—H8*WA*···O5*W*	0.85 (1)	2.02 (1)	2.856 (3)	171 (4)

Symmetry codes: (i) *x*−1/2, −*y*, *z*−1/2; (ii) *x*−1/2, −*y*+1, *z*−1/2; (iii) *x*−1, *y*, *z*; (iv) *x*, *y*−1, *z*; (v) *x*−1/2, −*y*, *z*+1/2; (vi) *x*+1/2, −*y*, *z*−1/2; (vii) *x*−1/2, −*y*+1, *z*+1/2.
